# The intra-observer reproducibility of cardiovascular magnetic resonance myocardial feature tracking strain assessment is independent of field strength

**DOI:** 10.1186/1532-429X-15-S1-E13

**Published:** 2013-01-30

**Authors:** Andreas Schuster, Geraint Morton, Shazia T Hussain, Roy Jogiya, Shelby Kutty, Kaleab N Asrress, Marcus R Makowski, Boris Bigalke, Divaka Perera, Philipp B Beerbaum, Eike Nagel

**Affiliations:** 1 Division of Imaging Sciences and Biomedical Engineering, King's College London, London, UK; 2 Department of Cardiology and Pneumology and Heart Research Center, Georg-August-University, Göttingen, Germany; 3 Joint Division of Pediatric Cardiology, Children's Hospital and Medical Center, University of Nebraska/Creighton University, Omaha, NE, USA; 4 Department of Radiology, Charite, Berlin, Germany; 5 Department of Cardiology, Eberhard-Karls-University, Tübingen, Germany; 6 Dept. for Radiology & Paediatric Cardiology, Radboud University Nijmegen Medical Centre, Nijmegen, The Netherlands

## Background

Cardiovascular magnetic resonance myocardial feature tracking (CMR-FT) is a promising novel method for quantification of myocardial wall mechanics from standard steady-state free precession (SSFP) images. We sought to determine whether magnetic field strength affects the intra-observer reproducibility of CMR-FT strain analysis.

## Methods

We studied 2 groups, each consisting of 10 healthy subjects, at 1.5 or 3 Tesla. Analysis was performed at baseline and after 4 weeks using dedicated CMR-FT prototype software (Tomtec, Germany) to analyse standard SSFP cine images. Right ventricular (RV) and left ventricular (LV) longitudinal strain (EllRV and EllLV) and LV long-axis radial strain (ErrLAX) were derived from the 4-chamber cine, and LV short-axis circumferential and radial strains (EccSAX, ErrSAX) from the short-axis orientation. Strain parameters were assessed together with LV ejection fraction (EF) and volumes. Intra-observer reproducibility was determined by comparison of the first and the second analysis in both groups.

## Results

In all volunteers resting strain parameters were successfully derived from the SSFP images. There was no difference in strain parameters, volumes and EF between field strengths (p>0.05). In general EccSAX was the most reproducible strain parameter as determined by the coefficient of variation (CV) at 1.5 Tesla (CV 13.3% and 46% global and segmental respectively) and 3 Tesla (CV 17.2% and 31.1% global and segmental respectively). The least reproducible parameter was EllRV (CV 1.5 T 28.7% and 53.2%; 3T 43.5% and 63.3% global and segmental respectively).

## Conclusions

CMR-FT results are similar with reasonable intra-observer reproducibility in different groups of volunteers at 1.5 and 3 Tesla. CMR-FT is a promising novel technique and our data indicate that results might be transferable between field strengths. However there is a considerable amount of segmental variability indicating that further refinements are needed before CMR-FT can be fully established in clinical routine for quantitative assessment of wall mechanics and strain.

## Funding

AS receives grant support from the British Heart Foundation (BHF) (RE/08/003 and FS/10/029/28253) and the Biomedical Research Centre (BRC-CTF 196). SK receives grant support from the American College of Cardiology Foundation, the Edna Ittner Pediatric Foundation, and the Children's Hospital and Medical Center Foundation. EN receives grant support from BHF (RE/08/003), the Wellcome Trust and Engineering and Physical Sciences Research Council (EPSRC, WT 088641/Z/09/Z) and the National Institute for Health Research (NIHR) via the comprehensive BRC award to Guy's and St Thomas' NHS Foundation Trust in partnership with King's College London.

